# Metabolites Identified during Varied Doses of *Aspergillus* Species in *Zea mays* Grains, and Their Correlation with Aflatoxin Levels

**DOI:** 10.3390/toxins10050187

**Published:** 2018-05-07

**Authors:** Titilayo D. O. Falade, Panagiotis K. Chrysanthopoulos, Mark P. Hodson, Yasmina Sultanbawa, Mary Fletcher, Ross Darnell, Sam Korie, Glen Fox

**Affiliations:** 1 International Institute of Tropical Agriculture, Headquarters and West Africa Hub, Ibadan 200001, Nigeria; s.korie@cgiar.org; 2 Queensland Alliance for Agriculture and Food Innovation, The University of Queensland Health and Food Sciences Precinct, Coopers Plains, Brisbane, QLD 4108, Australia; y.sultanbawa@uq.edu.au (Y.S.); mary.fletcher@uq.edu.au (M.F.); g.fox1@uq.edu.au (G.F.); 3 Metabolomics Australia, Australian Institute for Bioengineering and Nanotechnology, The University of Queensland, St. Lucia, Brisbane, QLD 4072, Australia; panos.chrysanthopoulos@ccrm.ca (P.K.C.); m.hodson1@uq.edu.au (M.P.H.); 4 Centre for Commercialization of Regenerative Medicine, Toronto, ON M1G 5M5, Canada; 5 School of Pharmacy, The University of Queensland, Woolloongabba, Brisbane, QLD 4102, Australia; 6 Commonwealth Scientific and Industrial Research Organisation, Ecosciences Precinct, Dutton Park, Brisbane, QLD 4102, Australia; ross.darnell@csiro.au

**Keywords:** aflatoxin, *Aspergillus parasiticus*, metabolomics, metabolite, *Zea mays*

## Abstract

Aflatoxin contamination is associated with the development of aflatoxigenic fungi such as *Aspergillus flavus* and *A. parasiticus* on food grains. This study was aimed at investigating metabolites produced during fungal development on maize and their correlation with aflatoxin levels. Maize cobs were harvested at R3 (milk), R4 (dough), and R5 (dent) stages of maturity. Individual kernels were inoculated in petri dishes with four doses of fungal spores. Fungal colonisation, metabolite profile, and aflatoxin levels were examined. Grain colonisation decreased with kernel maturity: milk-, dough-, and dent-stage kernels by approximately 100%, 60%, and 30% respectively. Aflatoxin levels increased with dose at dough and dent stages. Polar metabolites including alanine, proline, serine, valine, inositol, iso-leucine, sucrose, fructose, trehalose, turanose, mannitol, glycerol, arabitol, inositol, myo-inositol, and some intermediates of the tricarboxylic acid cycle (TCA—also known as citric acid or Krebs cycle) were important for dose classification. Important non-polar metabolites included arachidic, palmitic, stearic, 3,4-xylylic, and margaric acids. Aflatoxin levels correlated with levels of several polar metabolites. The strongest positive and negative correlations were with arabitol (*R* = 0.48) and turanose and (*R* = −0.53), respectively. Several metabolites were interconnected with the TCA; interconnections of the metabolites with the TCA cycle varied depending upon the grain maturity.

## 1. Introduction

*Aspergillus flavus* and *A. parasiticus* are major aflatoxin-producing *Aspergilli* that contaminate many agricultural products including maize, groundnuts, sesame, and sunflower [1,2,3], rendering them unsafe at elevated aflatoxin levels. High levels of aflatoxin exposure are associated with hepatic dysfunction, teratogenic defects, and immune suppression [4,5]. In children, stunting and reduced cognitive ability are linked with high levels of aflatoxin ingestion [6,7,8] although a direct relationship is not established. Aflatoxin accumulation occurs with organic (rather than inorganic) sources of carbon and nitrogen, with a preference for simple sugars, acidic conditions, and species-specific utilisation of amino acids [9,10,11], such as are present in food grains like maize (*Zea mays*). The same strains of *A. flavus* have been reported to produce disparate amounts of aflatoxins when on different substrates [12], suggesting differences in metabolic processes. Therefore, understanding the metabolic processes that affect the levels of aflatoxin accumulation is beneficial. There are suggestions that secondary and primary metabolism may be linked much more than previously thought. There is an associated metabolism of amino acids and ethanol (related to primary metabolism) in aflatoxigenic *A. parasiticus* during aflatoxin biosynthesis (related to secondary metabolism) [13]. Reports of metabolic changes in the maize crop with other pathogens [14,15] as well as in other plant-host relationships [16,17,18] have also been reported.

In recent years, metabolite studies have been conducted in maize plants exposed to abiotic stresses to provide information on affected cellular processes [19,20]. Metabolite variability is recorded in uncontaminated maize, with up to 75 metabolites differentially produced in 14 maize genotypes. Variations across genotypes decreased in the order lipids > amino acids > carbohydrates [21]. Abiotic stress factors are associated with differential metabolite concentrations in the shoots and roots of maize. Salt stress is associated with elevated levels of alanine and sucrose, but a decrease in glucose [20]. Alterations to the metabolite profile in the leaves of maize plants have also been observed with heat and drought stresses to the maize crop, with stressed plants exhibiting high sugar and amino acid levels [22]. Biotic stresses occur during crop infection and are also important for changes within the crop matrices. In crop-pathogen interactions, fungal metabolic pathways have been investigated using gene knock-out techniques [13,23,24].

Using comparative metabolomics studies, mutant strains have been compared with their wild type to understand the gene function via observation of the differential metabolite production. This has provided insight into the gene functions and the specific biochemical pathways in the studied environment. In a study of the global secondary metabolism in *A. parasiticus*, velvet (*veA*) gene mutation was identified to cause disruptions in branch-chained amino acids such as leucine, isoleucine, and valine [13]. In another gene knock-out experiment in *A. flavus*, probable polyketide synthase27 (*pks27*) was identified as responsible for encoding a gene with homology to the aflatoxin cluster polyketide synthase, and important metabolites were related to building blocks for malonyl-coA [23]. Furthermore, when the pathways of the LaeA-regulated clusters (*lna* and *lnb)* were investigated, a reduction in l-tyrosine was found to be differential [24]. The LaeA-regulated cluster (*lna* and *lnb*) genes were also found to be important for sclerotia formation in the *A. flavus*. These studies of metabolites continue to be valuable. Metabolites provide diverse yet interrelated information for cellular functions. Primary and secondary metabolites can, therefore, provide a means to understanding and deciphering cellular processes. These processes may involve genetic function, protein function, as well as regulatory and signalling processes in the cell [13,14,25].

Metabolomics typically utilize chromatography and mass spectrometry platforms to perform targeted and/or non-targeted analyses. Metabolomic profiling offers an opportunity to gain understanding of metabolite changes caused by aflatoxigenic fungi infection, as it is at present unclear how metabolites differ in maize grains contaminated with aflatoxigenic fungi. In this paper, we report metabolite profile differences in maturing maize grains (at R3 (milk), R4 (dough), and R5 (dent) reproductive stages) inoculated with different doses of an aflatoxigenic fungus using untargeted metabolomics as well as targeted assessment of aflatoxin levels. This was done because contamination of maize kernels has been reported from the milk stage of kernel development [26,27]. The maize grains were from plants grown in a glasshouse.

## 2. Results

### 2.1. Crop Development

**Figure 1:**
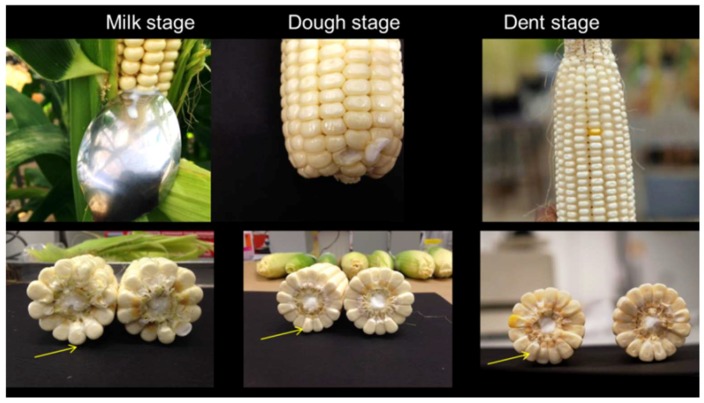
Maize cobs at milk, dough, and dent stages of reproductive development.

Maize plants grown in the glasshouse were taller and thinner than field grown crops of the same variety (stem girth was about 2 cm stem diameter and plant height was >3 m high). The taller and thinner appearance was plausibly due to the limitation in solar intensity reported to affect plant height [28]. At the vegetative stage, seed emergence and crop development up to the V1 stage (at sign of first visible leaf collar) of development was satisfactory. However, at the V2 stage (at sign of second visible leaf collar), signs of copper deficiency were observed. This was attributed to the high organic content of the potting mix [29]. Therefore, the potting mix was slightly leached and two percent (2%) copper sulphate was applied as foliar fertilizer *ad libitum*. The crop recovered within a week and development was good for the duration of the plants vegetative and reproductive stages of growth. Crop development at the reproductive stages were satisfactory with R1 (silk), R2 (blister), milk (R3), dough (R4), and dent (R5) stages of crop development reached at 68, 76, 90, 97, and 105 days after planting (DAP), respectively. Stages were identified based on endosperm consistency, milk line position (arrows in milk and dough stage kernels), and signs of starch accumulation in the kernels (arrow in dent stage kernels) (Figure 1). Grain filling was ideal.

### 2.2. Grain Colonisation with Fungal Spores

**Figure 2:**
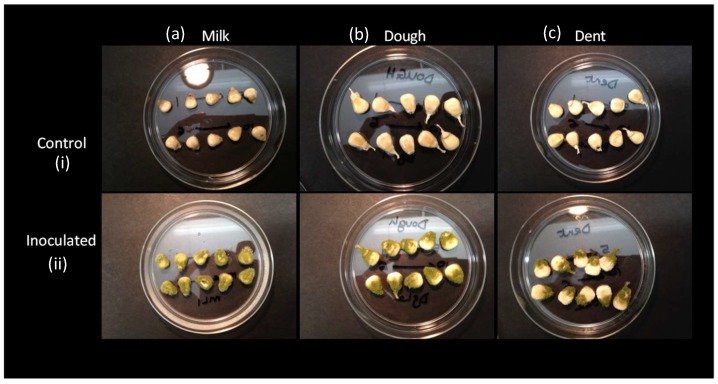
Grain colonisation (**a**) milk, (**b**) dough, and (**c**) dent kernels (i) control and (ii) inoculated.

The control, low, medium, and high doses of fungal suspensions had visual differences in suspension turbidity (not shown). However, at the end of the colonisation period within each grain maturity stage, there were not visually observable differences of fungal dose, except for the control samples. The colonisation score for the control samples at all stages of maturity was 0 (0%). However, across the grain maturity stages, the differences in extent of grain colonisation were observed at the end of the 72-h incubation period. Kernels from the milk, dough, and dent stages had grain colonisation scores of 3 (100%), 2 (60%), and 1 (30%), respectively (Figure 2). Colonisation appeared to have commenced from the germ and progressed upward towards the upper part of the kernel. This was particularly noticeable in the pericarp of the dough stage kernels and the endosperm and pericarp of the dent stage kernels. However, the milk stage kernels were almost entirely covered by the fungal spores (Figure 2). The easily metabolisable simple molecules in the milk stage kernels and the less developed pericarp may have been responsible for the 100% colonisation of the kernels. The dent stage kernels on the other hand had the least proportion of the simple molecules, particularly in the endosperm. Colonisation pattern also agreed with this, as the starch portion accumulated towards the top of the kernel was the least colonised by the fungus (Figure 2).

### 2.3. Aflatoxin Levels

After the 72-h incubation period, aflatoxin levels (log AFT) ranged from 0.0 to 5.0. The fungi had progressed well in their metabolism and reached their active stage of aflatoxin production [30,31]. The range of absolute levels (log Absol) in all the kernels was from 0.0 to 4.3. Aflatoxins were detected in all the low, medium, and high dosed maize kernels (Table 1 and Table 2). There were no aflatoxins detected (less than LOD) in any of the control samples (Table 1). The inoculated samples had statistically higher aflatoxin levels (*p* < 0.05) than the control samples. This was true for both log AFT and log Absol (Table 1 and Table 2).

The inoculated samples had statistically higher aflatoxin levels (*p* < 0.05) than the control samples (Table 1). The range of log AFT in all the kernels was from less than 0.0 to 4.4. Within the milk stage, log AFT and log Absol in the low, medium, and high doses were not significantly different from one another (*p* > 0.05). (Table 1 and Table 2). However, within the dough and dent stage kernels, aflatoxins levels at the medium and high doses were statistically higher (*p* < 0.05) than those at the low dose (Table 1 and Table 2).

At low fungal dose, the aflatoxin levels were similar regardless of the stage of maturity (Table 1 and Table 2). At medium and high fungal doses, only the dough stage had statistically higher aflatoxin concentrations than the milk and dent stages, but absolute aflatoxin levels were higher at dough and dent stage than at milk (Table 2). The germ is more mature at the dough and dent stages and this perhaps explains the higher levels compared to the milk stage kernels.

### 2.4. Polar Metabolites Identification

#### 2.4.1. Stepwise Discriminant Analysis

Stepwise discriminant analysis (SDA) identified 27/141 metabolites as important for fungal dosage classification with above 70% accuracy (Table 3). From the 27 polar metabolites, 15 (56%) could be putatively identified using the 70% similarity match in the NIST MS library as indicated in the methods section, two metabolites (turanose and trehalose) had a 50% similarity match, while others were classified as unidentified. The major classes of the compounds identified were amino acids, sugars, and sugar alcohols. Specifically, these compounds were putatively identified as phosphoric acid, alanine, proline, serine, malate, gamma-aminobutyric acid (GABA), arabitol/xylitol, inositol, glyceryl-glycoside. Four compounds were identified generally as sugars (based on the large numbers of identification hits for sugars) (Table 4). The highest classification accuracy/least error was with the high dose (96%/0.04) while the lowest classification accuracy/highest error was with the control dose (73%/0.27) (Table 3).

Overall, the most abundant compounds detected in the kernels were arabitol/xylitol and malate. They had the highest relative abundance above 4 at the four dose levels. Proline was the most abundant amino acid detected (Table S1). Arabitol/xylitol levels increased, while proline levels decreased relative to control. Generally, metabolite levels declined in inoculated classes compared to control samples (Table S1). However, this trend reversed with two alcohols (inositol and arabitol/xylitol), two unidentified sugars (sugar-P252, sugar-P481), and two unidentified compounds (UK-P73 and UK-P74) in the inoculated classes. The correlation of the polar compounds to aflatoxins are presented in Table 4.

#### 2.4.2. Linear Discriminant Analysis

**Figure 3:**
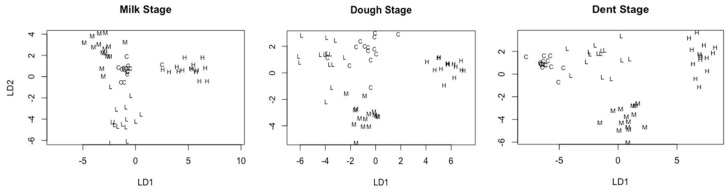
Cluster analysis of fungal dose in kernels using polar metabolite data at milk, dough, and dent stages of maturity. (C, L, M, and H represent control, low, medium, and high fungal doses). LD1 and LD2 are linear discriminant functions 1 and 2.

**Figure 4:**
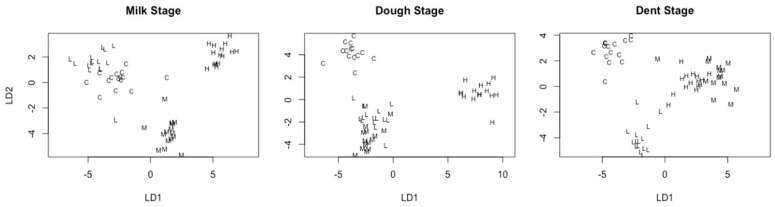
Cluster analysis of fungal dose in kernels using non-polar metabolite data at milk, dough, and dent stages of maturity (C, L, M, and H represent control, low, medium, and high fungal doses). LD1 and LD2 are linear discriminant functions 1 and 2.

Dose clusters utilising the identified metabolites demonstrated the ability of these metabolites for separation of doses. The most distinct clusters are seen in the milk and dent stages while the control and low doses of kernels inoculated at the dough stage were more loosely clustered (Figure 3 and Figure 4).

Generally, the classes of compounds of those identified were sugars, amino acids, and sugar alcohols (Figures S1–S12). The milk stage compounds useful for dose discrimination were 1,2-propanediol, alanine, aminomalonic acid, arabitol/xylitol, citric acid, d-erythro-pentitol, erythronoic acid, fructose, GABA, glyceric acid, glycerol, glycine, inositol, isoleucine, l-threitol, malonic acid, mannitol, myo-inositol, phosphoric acid, proline, serine, succinate, threonine, trehalose, turanose, uracil, valine, 14 unnamed sugars, and 37 compounds that could not be identified from the available databases. Among the unidentified compounds, UK-P69 increased with dose, whereas UK-P725, UK-P733, and UK-P44 decreased with dose increase. Fructose and an unidentified sugar-P190 were the most abundant in the milk stage samples. However, their patterns of accumulation were opposed to each other. While fructose decreased progressively with fungal dose, the levels of Sugar-P190 increased progressively with fungal dose (Figure S1a), possibly indicating a relationship with fungal dose. Unfortunately, the identity of this interesting sugar-P190 could not be elucidated from the available databases. Turanose also decreased with fungal dose. Among the alcohols, l-threitol, arabitol/xylitol and d-erythro-pentitol increased with fungal dosage, while mannitol and myo-inositol decreased with fungal dosage. Glyceric acid and uracil decreased with fungal dosage.

The dough stage compounds important for dose discrimination included 23 sugars, three amino acids, five alcohols, five acids, succinate, and malate. These compounds were putatively identified as trehalose, turanose, proline, serine, GABA, arabitol/xylitol, glycerol, inositol, l-threitol, myo-inositol, citric acid, erythronoic acid, glyceric acid, malonic acid, succinic acid, and malate. Among the sugars, sugars-P239, P602, fructose, and sucrose decreased with dosage, whereas, sugars-P190, P252, P551, and P639 increased with fungal dosage. Inositol, l-threitol, and arabitol or xylitol increased with fungal dosage, while myo-inositol decreased with fungal dosage.

The dent stage kernels useful for dose discrimination included 16 sugars, 11 amino acids (including α and γ amino acids), eight alcohols, nine acids, succinate, malate, glycine, uracil, and 45 unidentifiable compounds. Among the sugars, progressive decrease with dosage was observed with sugars-P245, P454, trehalose, turanose, sucrose, and fructose. Whereas, progressive increase was observed with sugars-P252, P320, P481, and disaccharide-P582. Amino acids threonine, alanine, proline, serine, valine, and isoleucine decreased progressively with fungal dosage. Among the alcohols, progressive decrease with dosage was observed with myo-inositol and 1,2-propanediol, while a progressive increase with dosage was observed with mannitol, glycerol, l-threitol, and inositol. Citric acid increased progressively with dosage while glyceric acid and erythronoic acid decreased progressively with dosage. The unidentified compounds are presented in the supplementary material but are not discussed further.

## 3. Discussion

### 3.1. Sugars

The sugar class made the highest contribution of compounds for dose classification at milk, dough, and dent stages of physiological maturity. Generally, there were much more elevated levels of sugars in the control kernels compared to the inoculated kernels at the milk stage of maturity. However, sugar-P190 increased with fungal dose at all stages of kernel maturity. Sugar-P454 was prominent at all stages and decreased with fungal dose. The trend of decreased sugar levels with increased fungal activity in the samples is consistent with the findings of others that sugar uptake is essential for fungal development and aflatoxin accumulation [32]. Sugars may be a trigger for aflatoxin accumulation [10]. The decreased levels of these sugars suggest their utilisation as carbon sources for biochemical processes, including for aflatoxin accumulation, as has been reported by others [32,33]. Fructose, glucose, and sucrose in peanuts are directly correlated with aflatoxin B1 in peanuts [34].

Fructose and turanose were identified as important for dose discrimination at the three maturity stages and had higher levels in the control samples compared to the inoculated samples. The use of fructose for aflatoxin accumulation has been corroborated by others [35] where they reported that fructose induces aflatoxin production between 24 h and peaks at 96 h (but induction is not excluded after 96 h). Therefore, the role of fructose is in both the exponential and stationary growth phases, i.e., before and after 96 h, respectively. Sucrose and trehalose were important in dough and dent stages, but not the milk stage, and decreased with increased fungal activity (Figures S4–S6). Sucrose levels increase in the germ as it matures [36]. This suggests that this simple sugar would be more accessible in the more mature dough and dent kernels. This explains why this sugar may be more relevant for dose selection in the dough and dent kernels, but not the milk-stage kernels. The preferential sucrose and lipid distribution in the germ may be contributory to this colonisation pattern. Lipids in the germ are suggested to be important for aflatoxin accumulation [37], and high sucrose levels in the germ are related to the metabolism of fatty acids [36]. This is an indication that fatty acids and sugars play roles that may correlate with aflatoxin biosynthesis.

There was significant negative correlation (*p* < 0.05) of fructose, sucrose, and turanose levels with aflatoxin concentrations (*R* = −0.25, −0.30, and −0.53, respectively).

The selection of turanose for aflatoxigenic fungi is in agreement with the reports of others where turanose is associated with virulence [38] and activation of mitogen-activated protein kinases (MAPKs) [39] in the MAPK pathway. Kinases are important for the transport of phosphates. Therefore, the identification of turanose in the current study is insightful, as phosphoric acid was also identified as a dose discriminating factor (Table S1). Studies from other researchers also suggest that turanose activates an extracellular invertase (Lin6) [39]. This is insightful as under conditions of stress, the maize cell reduces its biosynthesis of complex molecules of starch and instead expresses genes that increase the free hexoses in the cell by the action of invertases [27]. This suggests that the perception of turanose by the plant could be a stress stimulus signalling the plant to also increase its own invertase activity. The stronger correlation coefficient of turanose compared to fructose in relation to aflatoxins suggests that turanose plays a critical function in the secondary metabolism leading to aflatoxin biosynthesis.

Trehalose was important in dose identification in the dough and dent samples and significantly correlated (*p* < 0.05) with aflatoxins (R = −0.49) (Table 4). Trehalose is important for stress tolerance and viability of spores as an osmolyte in *A. flavus* and *A. nidulans*, which are close relatives of *A. parasiticus* (the isolate used in this study). Trehalose is a storage metabolite that increases within contaminating *A. flavus* after 48 h [40] and is required for stress tolerance in *A. nidulans* [41,42]. Although *A. nidulans* is not an aflatoxin-producer, it produces sterigmatocystin, which is a precursor to aflatoxins, and its biosynthetic pathways are similar to that of aflatoxin producers, but are truncated before they reach the aflatoxin step [43]. Therefore, there could be some similarity in the utilisation pattern and role of trehalose in both species. This is suspected, especially as trehalose is reported to be an important osmolyte, required for spore survival [42,44], and is important under conditions of osmotic stress contributing to aflatoxin accumulation [45]. The absence of a trehalose biosynthetic gene (*tpsA*) has been reported to result in the loss of spore viability, succumbing to heat stress and H_2_O_2_ stress (oxidative stress) [41]. Small quantities of trehalose are found in plants for stress tolerance [46] as observed by the small levels of trehalose present in the control samples.

The key role of sugars in aflatoxin biosynthesis is supported by the prominence of the sugar cluster in the aflatoxin biosynthetic pathway [47] and increased gene expression in high carbohydrate medium [10]. Others also confirmed natural deletion of genes in the sugar utilization cluster (*nadA-hexA-glcA-sugR*) as one of the deletion patterns, among others, that contributes to the loss of aflatoxigenicity in *Aspergillus* species [48]. However, this is not the only pattern of deletion that causes loss of aflatoxigenicity in non-aflatoxigenic strains of *A. flavus* [48,49]. There is also a reported relationship between the expression of the sugar cluster genes and the aflatoxin pathway regulatory genes *aflR* and *aflS*. Increased expression of *glcA*, *hxtA*, *nadA*, and *sugR* (and aflatoxins) are higher when the transcription ratio of *aflR:aflS* regulatory genes is high [50].

### 3.2. Sugar Alcohols

In this study, arabitol/xylitol was positively correlated with aflatoxin concentrations (R = 0.48, *p* < 0.05) (Table 4). Arabitol/xylitol was present in high relative amounts, above 4, while inositol amounts were below 0.5 (Table S1). The increase in the levels of arabitol is in agreement with reports of increased levels of arabitol levels as aflatoxin accumulated in cottonseed [40] and in fungal biomass of both *A. flavus* and *A. parasiticus* [51]. Others report that arabitol and aflatoxin B1 production co-occur [52]. These earlier reports, associated with both fungal biomass and aflatoxin accumulation, suggest that increased levels of arabitol may be related to both the primary and secondary metabolic pathways of the fungi. Arabitol has an osmoregulatory function [51], and this attribute is vital for coping with conditions of osmotic stress, which is a predisposing factor for aflatoxin accumulation [53,54]. Information about xylitol and aflatoxins or aflatoxigenic fungi is sparse. However, there has been mention about the inability of xylitol to induce aflatoxins [55]. It is, therefore, more likely that this putatively identified compound is arabitol rather than xylitol. However, empirical verification is still required with chemical standards to certify the identity of this metabolite.

In the current study, mannitol levels correlated with aflatoxins (R = −0.16, *p* = 0.05) (Table 4). Mannitol is important for stress tolerance [44], where mannitol may be temporarily enabling cells to maintain their integrity during stress [40]. Like arabitol, mannitol also plays an osmoregulatory function. This has been reported in both aflatoxigenic and non-aflatoxigenic fungi of *Aspergillus* spp. [40]. Some authors report that mannitol production occurs concurrently with aflatoxin accumulation in maize [52], others report that its accumulation occurs regardless of toxigenicity potential [51]. In this study, glycerol is another important sugar alcohol that significantly correlated with aflatoxins (R = 0.41, *p* < 0.05) (Table 4). Being an osmoprotectant, glycerol maintains cellular integrity by protecting the cell against damage due to dehydration, heat, or free radicals [44]. Protection by this sugar alcohol prevents macromolecular and enzymatic damage. Glycerol levels have also been reported to increase under conditions of water stress in *A. flavus* and *A. parasiticus* [51]. Accumulation of glycerol could suggest increased activity of cellular processes that lead to increased tricarboxylic acid (TCA) cycle metabolic processes. Also, others report that glycerol stimulates the synthesis of aflatoxins [56].

Inositol and myo-inositol are cellular signalling moieties [57]. Signalling pathways are critical in *A. flavus* colonisation in the maize kernel [58]. Inositol and myo-inositol statistically correlated (*p* < 0.05) with aflatoxins (R = 0.45 and −0.47, respectively). Their patterns of accumulation in the infected kernels were contrasting. Inositol levels increased, while myo-inositol levels decreased (Figures S12–S14). Their converse relationship suggests different kinds of cellular processes occurring within the kernel with increased fungal dose.

### 3.3. Polar Acids

The other identified metabolites will be discussed in two groups: (1) amino acids and (2) other acids and uracil.

#### 3.3.1. Amino Acids

Analysis of all doses showed that amino acids were generally higher in the control samples than in the inoculated samples (Figures S1–S3), suggesting catabolism of these amino acids for fungal metabolism. The levels of amino acids identified significantly correlated (*p* < 0.05) with aflatoxin levels: alanine (R = −0.41), proline (R = −0.48), serine (R = −0.42), gamma-aminobutyric acid (GABA) (R = −0.39), valine (R = −0.45), threonine (R = −0.44), iso-leucine (R = −0.46), and glycine (R = −0.31) (Table 4). Threonine, serine, isoleucine, leucine, aspartic acid, and methionine have been observed to be influenced by *A. parasiticus* in groundnuts [59]. Similarly, during *A. parasiticus* metabolism, the catabolism of valine, leucine, and isoleucine have been detected [13]. In *A. flavus*, increase in aflatoxins has been reported with catabolism of leucine and iso-leucine [60]. Proline has been reported as an amino acid utilised by aflatoxigenic species, and that triggers aflatoxin production [61]. However, there are opposing arguments as to the role of proline as either a scavenger of reactive oxygen species (ROS) or responsible for the accumulation of ROS in the cell [62,63]. Proline is also reported to accumulate in stressed plants [64] and is related to aflatoxin biosynthesis, suggesting an alignment between the stressed plant and the invading fungus. It is probable that the fungus may have developed this as an adaptation strategy, such that elevated levels of proline may be a signal to the invading fungus, thus triggering increased metabolism. In *A. niger* (a close relative of aflatoxigeic species *A. flavus* and *A. parasiticus*), there is a positive relationship between proline and alanine and the stimulation of conidia germination [65]. High levels of aflatoxin production supported by proline have been reported [61], and increased catabolism of alanine has been detected to match increased accumulation of aflatoxins [60]. Furthermore, alanine is a carbon source for fungal primary and secondary metabolism [30,66], and a stimulant for aflatoxin accumulation [67]. GABA is not an alpha amino acid but was important for dose classification at the three stages of grain maturity. This amino acid is a signalling molecule produced by plants and biological systems for stress response [68,69] and is important for enabling the maize plant to adjust to stress [64], also, it is a source of nitrogen for pathogenic fungi [69]. Pathogenic fungi stimulate GABA production in plant cells and exploit GABA for their own development [69]. GABA’s involvement in the production of succinate in the TCA cycle [70] via the GABA shunt is well known. In some instances, GABA is incorporated in fermented meals (including the use of *A. oryzae* for fermentation) for functional attributes [71]. Being desirable in foods could lead to breeding for GABA enrichment, which should proceed with caution as GABA levels are significantly (*p* < 0.05) correlated with aflatoxin levels. Another amino acid of importance in this study was aminomalonic acid, however, it is scarcely studied with respect to aflatoxigenic fungi. It is an unstable amino acid [72]. It has been suggested that aminomalonic acid is a constituent of proteins (although not formed from alpha amino acids,) and its production may be caused by synthesis errors or oxidative damage in proteins [73].

#### 3.3.2. Other Metabolites

The levels of erythronic acid in the control samples were higher than those in the inoculated samples, and erythronic acid levels were significantly correlated (*p* < 0.05) with aflatoxins (R = −0.41) (Table 4). It has been suggested that erythronic acid may be formed from glycated lysine residues or the breakdown of ascorbic acid [74,75]. Accumulation of antioxidants such as ascorbic acid and glutathione polyphenols help the plant in antagonistic measures against antioxidant species that would interfere with membrane integrity. Antioxidants can reduce aflatoxin production [76]. Therefore, antioxidant breakdown would be necessary for continued aflatoxin accumulation, but the lower levels of erythronic acid in inoculated samples suggest a different explanation.

In this study, aflatoxin levels correlated with malonic acid, glyceric acid, and uracil (R = 0.17, −0.40 and −0.39, respectively; *p* ≤ 0.05), as well as phosphoric acid with R = 0.21 (*p* < 0.05) (Table 4). Malonic acid is responsible for the stimulation of acetate incorporation to aflatoxin at low concentrations [77], therefore, this supports this correlation. While the relationship of glyceric acid specifically to aflatoxins is sparsely reported, it is known that glyceric acid is formed from the oxidation of glycerol, and glycerol accumulates in the cells of *A. flavus* and *A. parasiticus* under conditions of osmotic stress [51]. The relationship between uracil and aflatoxin production may be due to increased colonisation and thus RNA production and turnover.

Levels of phosphoric acid, citric acid, and malate significantly correlated with aflatoxins (R = 0.21, 0.36, 0.18, and 0.21, respectively, *p* < 0.05), but not succinate (Table 4). The acids or ions of citric acid, succinic acid (ion is succinate), malic acid (ion is malate), and phosphoric acid (ion is phosphate) are intermediates in the tricarboxylic acid cycle. Phosphate groups are important in many cellular metabolic processes. The detection of phosphoric acid for dose classification suggests changes in cellular activity involving kinases in phosphorylation. Within the glycolytic and tricarboxylic acid pathways, the phosphate groups feature extensively for energy transfer and generation in the adenosine diphosphate (ADP) and adenosine triphosphate (ATP) moieties.

The use of these TCA intermediates in dose classification and their significant correlations, suggest that the TCA cycle plays a critical role in fungal dose and aflatoxin accumulation. However, both aflatoxin and non-aflatoxin-producing strains produce citric acid. *A. niger* is an efficient producer of citric acid [78], and is used for commercial citric acid production. However, aflatoxigenic species are stimulated by citric acid to undergo an additional step of conversion of acetyl coA into malonyl coA for aflatoxin biosynthesis [30]. Since this conversion requires stimulation by citric acid, it suggests that citric acid utilisation is a crucial link between the primary and secondary metabolic pathways within aflatoxigenic species. The exponential growth stage leads to pyruvate accumulation. However, accumulation of pyruvate (product of glycolysis) is toxic to the fungus, so the fungus needs to synthesize other molecules from pyruvate, thus driving TCA precursors into sustaining the cycle and the secondary metabolic reactions (where genetically possible). Acetate is the primary molecule in the aflatoxin biosynthetic pathway [43] and in the TCA cycle [79]. This confirms an interrelationship between the TCA cycle and aflatoxin biosynthesis. Furthermore, the biochemical processes of the TCA cycle occur in the mitochondrion, and aflatoxin-producing fungi have distorted mitochondria and accumulate ethanol [13,60]. Ethanol formed from fermentation of sugars could be an indication that the fermentation of sugars is necessary for the biosynthesis of aflatoxins.

#### 3.3.3. Fatty Acid Methyl Esters (FAME) Identification

None of the FAME levels had significant correlations (*p* > 0.05) with aflatoxin levels. However, clusters were not as distinctly separated as with the polar metabolites (Figure 4). In milk and dough kernels, the levels of putatively identified 2,4-Di-*tert*-butylphenol metabolites were much lower at the high dose than at other doses, but this pattern was not observed in dent-stage kernels (Figures S10–S12). There are suggestions that this phenolic compound is protective against pathogens such as *A. niger*, *F. oxysporum*, *Phytophthora capsici*, *P. chrysogenum*, and *Colletotrichum* sp. [80,81,82] and has allelopathic abilities on microbial biomass in soils [83]. There is little evidence that maize kernels may produce this compound, or if it is antagonistic to aflatoxigenic *Aspergilli*. Nevertheless, in a study to investigate the action of this metabolite on cell lines from a rat, 2,4-Di-*tert*-butylphenol was reported to have antioxidant activity comparable to vitamin C, particularly against hydrogen peroxide-induced oxidative stress [84]. Thus, it could be an antioxidant protectant related to aflatoxin accumulation, but this requires confirmation.

Arachidic acid is a saturated fatty acid present in corn oil and peanut oil [85,86]; it is related to elevated aflatoxins and delayed peanut harvest. [87]. Its production by *A. flavus* and *A. parasiticus* grown on malt extract agar has been reported [88]*,* as has its presence in plant pathogens including *A. flavus*, *A nidulans*, *A. niger*, and *F. moniliforme* (now termed *F. verticillioides*) [88,89,90]. Palmitic acid and margaric acid (saturated fatty acids present in corn oil [85,86]) are reported to be antagonistic to *Fusarium* sp. [91] but not *A flavus* and *A. parasiticus* [88]. Therefore, increased production of fungus-specific metabolites such as palmitic acid could favour aflatoxin-producing organisms, but not fumonisin-producing organisms. Palmitic acid supports aflatoxin production [92,93,94] and is not inhibitory to *A. flavus* and *A. parasiticus* [88]. However, delayed harvest in peanuts is associated with higher levels of palmitic acid and increased aflatoxin levels [87]. Stearic acid is a fatty acid present in the maize kernel [95,96], and it is reported to stimulate the growth of *A. flavus* and *A. parasiticus,* aflatoxin production [93,97,98], and to be attractive to insect pests and pathogens of maize [99]. Information about the roles of gondoic and xylylic acids with respect to aflatoxigenic fungi is sparse. Margaric acid has been detected in wheat [100]; it is reported to be absent in maize [101,102]. However, it was putatively identified in these samples, including the controls, therefore, specific identification of this metabolite is required for confirmation of its incidence. Unfortunately, metabolites that had levels that were significantly correlated (*p* < 0.05) with aflatoxins were among the unidentified samples. These were F3 (R = 0.20), F25 (R = 0.21), F28 (R = 0.19), F31 (R = 0.19), F34 (R = 0.20), F90 (R = 0.23), F91 (R = 0.21), F93 (R = 0.18), and F109 (R = 0.22).

Elevated levels of sugars and beta-oxidation of lipids are important triggers for aflatoxin stimulation. Sugar levels increase due to the breakdown of starch by *Aspergilli* and increased starch catabolism and invertases activity by maize in defence against fungal invasion. As accumulations of products of glycolysis are toxic to the fungus, glycolytic pathways need to be consumed. The TCA cycle provides an outlet for products of glycolysis. Increased transcription of genes relevant for glycolysis and the tricarboxylic acid cycle, an increase in free hexose content, and a decrease in starch content have also been observed in biotic-stressed maize. The aflatoxin biosynthetic pathway supports the role of sugars as the aflatoxin gene cluster has an 80-kb region containing at least 25 open reading frames (*aflA* to *afl* Y) involved in aflatoxin biosynthesis and an untranslated sugar cluster region.

Aflatoxin production is stimulated by oxidative stress induced by radical producers, while in the absence of stress, fungi lose ability for sclerotia production and thus aflatoxin biosynthesis. Although, the primary response of fungi to stress is not aflatoxin production but the production of antioxidants, aflatoxins are eventually synthesised in response to stimulation. Fatty acids from maize have been reported to stimulate the β-oxidation pathway in mitochondria of the fungi and contribute to aflatoxin biosynthesis [43,103,104]. The exact mechanism by which this stimulation occurs is not clearly elucidated. However, oxylipin (oxidized lipids) cross-talk between the host and the fungus might be important for aflatoxin biosynthesis with increased access to zinc promoting aflatoxin accumulation [105,106,107]. This suggests that the relationship with lipid oxidation is possibly associated with the unidentified non-polar metabolites in the current study that were significantly correlated with aflatoxins. While it is true that changes in primary metabolism are related to stress, the complexity involved in the defence pathways makes it challenging to link changes in primary metabolism to the secondary metabolism pathways that are associated with plant stress. Secondary metabolite production is not directly correlated with physiological importance in the host and is more an aspect of the plants’ strategy to cope with unfavourable ecological conditions to enhance survival. Nevertheless, these changes in primary metabolism affect carbon fixation pathways and osmotic adjustment that affect stress tolerance.

## 4. Conclusions

The findings in the current research identified sugars and amino acids as important for dose classification and correlation with aflatoxin levels. It is possible that the link is due to the catabolism of complex molecules such as carbohydrates, fats, and proteins, resulting in the production of acetyl coA. Acetyl coA is the starting molecule for both the TCA cycle and aflatoxin biosynthesis. During conditions of stress, an additional requirement by cells is to maintain membrane integrity. Metabolites including trehalose, mannitol, and sorbitol are important for osmo-protection and are related to the accumulation of the fungal toxin. The findings from this research, therefore, show changes in contaminated maize grains that can be investigated further in a targeted approach to confirm the putative identities of the metabolites identified and discussed here.

## 5. Materials and Methods

### 5.1. Crop Development

Maize seeds of 33v62 variety (Pioneer Overseas Corporation, Johnston, IA, USA) were planted at the University of Queensland’s (UQ) temperature-controlled glasshouse, St. Lucia, Brisbane. Three seeds were planted per pot in 36 eight-litre pots and later thinned down to one plant per pot. The UQ23 potting media was used, comprised of 70% composted pine bark, 30% coco peat, and augmented at 1 kg m^−3^ Yates flowtrace (Yates, Clayton, Australia), 1 kg m^−3^ iron sulphate heptahydrate, 0.4 kg m^−3^ superphosphate, 0.03 kg m^−3^ copper sulphate, and 1 kg m^−3^ gypsum [108]. Basal fertilizer application was done with Osmocote^®^ slow release fertilizer (Osmocote, Bella Vista, Australia) at planting. Another application was made post-emergence as a top dressing and again at 50% crop tasselling. After germination, plants were thinned to one plant per pot and watered daily. Crops were propagated in a temperature-controlled glasshouse at an average temperature of 26.2 °C ± 4 °C. Cobs were harvested at 90, 97, and 105 days after planting (DAP), corresponding to the R3 (milk), R4 (dough) and R5 (dent) stages of crop maturity [109]. Maize cobs were placed in labelled polythene bags in a 4 °C portable cooler box immediately after harvest, and samples were transferred to the laboratory. Uniformly aged kernels were detached between the 10th and 20th rows from the bottom of the cob. Ten kernels were detached. After detachment, kernels were frozen at −20 °C in preparation for lyophilisation.

### 5.2. Grain Colonisation with Fungal Spores

Grains were colonised using fungal doses of *A. parasiticus* F75, provided by the Queensland Department of Agriculture & Fisheries’ (QDAF) laboratory (Brisbane, Australia). Spores were prepared for grain inoculations by dispensing an 8 µL spore suspension from working culture on the centre of V8 juice media plates in 5 cm petri dishes. Inoculated petri dishes were incubated unilluminated at 25 °C for five days. At the end of the incubation period, fungal spores were used to prepare doses of fungal spore suspensions by gently rinsing off the surface of the agar plate with 0.1% Tween 80 solution. The fungal doses, namely control, low, medium and high doses, were achieved by rinsing spores from none, one, four, and sixteen 5-cm petri dishes to provide 9 mL Tween 80 solutions of each dose. Quantitative measurements of the spore concentrations were not taken during experimentation.

**Figure 5:**
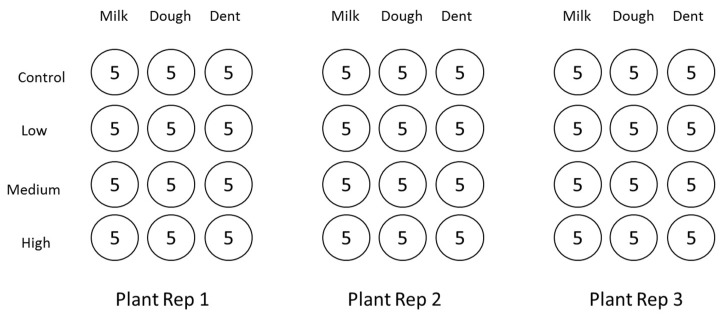
Experimental design layout.

Kernels of the milk, dough, and dent stages from the three plant replications were surface sterilized with 70% alcohol and rinsed in three changes of sterile distilled water. Sterile kernels were completely immersed in the respective dose suspensions (control, low, medium, and high) for 1 min contact time for fungal inoculation of the kernels. After fungal attachment, ten inoculated kernels from each replication were placed in sterile 9 cm petri dishes in a 4 × 3 factorial design with three replicates. Out of the 10 kernels, five were retrieved for analysis and the additional five were included in the event of a loss during experimentation, but were later discarded. Total number of kernels used for experiments was 180 (i.e., 4 × 3 × 3 × 5). A layout of the experiment is illustrated in Figure 5. Colonised kernels were incubated unilluminated at 25 °C for 72 h. At the end of the incubation period, kernels were scored for fungal colonisation by visual observation. Percentage grain colonisation (colonisation score) were assigned as follows: 0% (0), 1–30% (1), 40–60% (2), and 70–100% (3) based on surface area visibly covered by fungal growth. After visual scoring, kernels were frozen at −20 °C to halt fungal activity and in preparation for lyophilisation.

### 5.3. Chemical Analyses

#### 5.3.1. Sample Preparation and Extraction for Metabolomics Analysis

Extracts for analysis of metabolites were from inoculated, freeze-dried individual kernels. Polar and non-polar extracts of the milled maize kernels were obtained using protocols modified from [110]. Adapted protocols were developed at Metabolomics Australia. Using a Retsch Mill (RMM 301, M, Retsch, Haan, Germany), kernels were crushed to powder by stainless steel balls at 30 Hz for 10 s in 10 mL steel jars. Cross contamination between milled kernels was prevented by decontaminating the stainless-steel jars and balls with 70% ethanol. After milling each kernel, 50 mg of ground sample was weighed into tared 2 mL microtubes. To each 2 mL-microtube containing 50 mg of sample, 600 µL of methanol/chloroform [2:1 (*v*/*v*)] was added. The methanol/chloroform solution contained internal standards consisting of 1 mM stock ^13^C_6_ Sorbitol + 10 mM stock ^13^C_5_, ^15^N Valine and 5 mM ^13^C myristic acid. The milled grain/solvent mixture was vortexed, after which 200 µL chloroform was added and was mixed again with a vortex mixer. Thereafter, the mixture was sonicated at 70 °C for 15 min. Following this, 400 µL of milliQ water was added and the suspension mixed again with a vortex mixer.

The polar and non-polar fractions were separated by centrifuging the mixture at 16,000× *g* (at room temperature) for 15 min. Polar and non-polar extracts were transferred by pipette into separate tubes. The polar extract was further extracted from the non-polar fractions by adding 300 µL chloroform, mixing with a vortex, and centrifuging at 16,000 × *g* for 10 min. The polar and non-polar phases were again separated, and the non-polar extracts were pooled together. Total extracted volumes ranged from 400 to 600 µL. From each extract, 50 µL in glass inserts were placed in open labelled micro tubes and dried using the V-AQ program of a vacuum concentrator without heating (Concentrator Plus, Eppendorf, Germany). Dried extracts were capped in the micro tubes and stored upright in a silica gel/desiccator at room temperature prior to analysis. In addition to the samples, a pooled sample was also prepared and similarly dried. This pooled sample was for quality control (QC) purposes [111]. The total number of extracts was 179 because one kernel (a control sample at the dough stage of maturity) was lost during sample extraction. Following sample extraction, the polar and non-polar phase analytes were separated by gas chromatography and detected via tandem mass spectrometry.

#### 5.3.2. Polar Phase GC-MS Metabolomics Analysis

Derivatisation and introduction of the samples was performed using a Gerstel MPS-2XL autosampler equipped with a heated agitator (Gerstel GmbH & Co. KG, Mülheim an der Ruhr, Germany). Each sample was derivatised to its methoxy-TMS derivatives through a one hour reaction with 10 μL of 40 mg/mL methoxyamine hydroxychloride solution in pyridine (Sigma-Aldrich, St. Louis, MO, USA), followed by a two hour reaction with 20 μL of *N*,*O*-bis-(trimethylsilyl)-trifluoroacetamide (BSTFA) with 1% trimethylchlorosilane (TMCS) (Macherey-Nagel GmbH & Co. KG, Düren, Germany); both reactions were at 37 °C under constant stirring. Derivatised samples (1 μL) were then injected in splitless mode, at 250 °C using helium as a carrier gas under a constant flow of 0.718 mL/min. Metabolites were separated on a Varian capillary column (Factor FOUR VF-5 ms: 0.25 mm i.d., 0.25 μm film, 30 m length with a 10 m fused guard column; Varian, Mulgrave, Australia) installed on an Agilent 7890A gas chromatograph coupled to an Agilent 5975C MSD mass spectrometer (Agilent Technologies, Santa Clara, CA, USA). The initial temperature of the separation program (70 °C) was held for 1 min, then increased to 325 °C at a rate of 7 °C/min and held at 325 °C for 3.5 min. The ion source, quadrupole, and transfer line temperatures were set at 250 °C, 150 °C, and 280 °C, respectively. Metabolites were detected in total ion count (TIC) “scan” mode with scanning range 50–600 *m*/*z* at a rate of 2.66 scans/s. The acquisition order was randomized, and the samples were run with sample- and analyte-relevant calibration standards and pooled QC samples [111,112] intercalated after each block of 10 samples to control for reproducibility of data acquisition and to ensure data integrity. The instrument was cleaned and tuned prior to acquisition. This was conducted by Metabolomics Australia.

#### 5.3.3. Non-Polar Phase GC-MS Fatty Acid Methyl Ester (FAME) Metabolomics Analysis

Fatty acids in the non-polar phase were converted to their methyl esters and measured using a method developed for GC-MS by Metabolomics Australia. Briefly, lipids were saponified for 2 h at 80 °C by 200 μL of 2 M NaOH and 400 μL of methanol. After acidification with 40 μL of 37.5% HCl, 400 μL of chloroform were added and the suspension extensively vortexed. Phases were separated by centrifugation for 3 min at 3000× *g*. The layer of chloroform was collected and evaporated using the V-AQ program of a vacuum concentrator without heating (Concentrator Plus, Eppendorf, Germany). The dried chloroform extract was then treated with 200 μL of 2% H_2_SO_4_ in methanol and incubated for 2 h at 80 °C. The suspension was then cooled, 200 μL of 0.9% NaCl added, and vortexed thoroughly. The fatty acid methyl esters (FAMEs) were recovered in 300 μL of hexane, and 2 μL of the hexane layer was injected directly in the GC-MS in splitless mode, at 350 °C using helium as a carrier gas under a constant flow of 1 mL/min. Metabolites were separated on the Agilent GC-MS system as previously described. The initial temperature of the separation program (70 °C) was held for 5 min then increased to 320 °C at a rate of 9 °C/min and increased to 325 °C at 30 °C/min where it was held for 6.3 min (this temperature program was done to flush the column at maximum temperature). This section was conducted by Metabolomics Australia.

#### 5.3.4. GC-MS Data Processing

GC-MS metabolite peak identification was based on (a) an in-house library of standards that at the time of analysis consisted of about 750 unique compounds and (b) on the commercially available NIST MS library (2012) at 70% similarity match. A Retention Index (RI) ladder was also built using the following alkane compounds: dodecane, pentadecane, nonadecane, docosane, octosane, dotriacosane, and hexatriacosane. The RI values of the standard compounds were recalculated and adjusted based on the updated retention times of the ladder. RI values are constantly recalculated for accuracy. The hydrocarbons dissolved in hexane were injected in the beginning of the sequence. Pre-processing of GC-MS data was performed using AMDIS (Automated Mass Spectral Deconvolution and Identification System version 2.65, NIST, Gaithersburg, MD, USA) software for peak de-convolution and peak integration. The deconvolution parameters were selected as follows: component width = 6; adjacent peak subtraction = 2; resolution = medium; sensitivity = medium; shape requirement = medium. The data extracted from AMDIS were further processed using MassHunter Quantitative Analysis software for peak curation (version B.06.00, Agilent Technologies, Santa Clara, CA, USA) and Mass Profiler Professional software (version 12.1, Agilent Technologies, Santa Clara, CA, USA). All data were normalized to the internal standard (IS) intensity and were aligned with a retention time tolerance of 0.1 min. Once processed, the data matrix was normalised to sample weight and then exported in .csv format for external data analysis. Relative quantification of the metabolites was achieved by calculating the ratio of the metabolites’ peak areas to the internal standard’s peak area: sorbitol, valine, and myristic acid for sugars/alcohols, amino acids, and non-polar fractions, respectively. This section was conducted by Metabolomics Australia. The ratios were then analysed using multiple statistical analyses as follows.

#### 5.3.5. Extraction and Analysis of Aflatoxins

Samples were analysed for aflatoxin concentration (ng/g) and absolute aflatoxins levels (ng/kernel) in 140/179 kernels. The remaining 39/179 samples (a subset of milk stage kernels) were not included in aflatoxin analysis as there was insufficient milled sample remaining after extraction for metabolomics analysis. The aflatoxin concentrations were determined using UPLC-MS/MS [113]. Briefly, a maximum of 0.3 g milled kernels was mixed with 1.5 mL extraction solvent acetonitrile/MQ water/formic acid (790/200/10) in micro tubes. Samples were agitated for 10 min and centrifuged at 18,000× g. Extracts were filtered (using 0.2 μm PVDF syringe filters) with mobile phase A (water, formic acid, 99/1 *v*/*v* and 10 mM ammonium formate) in a 1:1 dilution and analysed.

### 5.4. Statistical Analyses of Aflatoxin and Metabolite Levels

For the metabolomics data, two data sets, consisting of polar and non-polar data, from the metabolite analysis were analysed statistically in two analytical procedures. The first procedure was a step-wise discriminant analysis (SDA) used to identify the important variables useful for classification of samples into the different dose groups of control (low, medium and high) regardless of the stage class. In the SDA procedure, the goal was to identify the most important independent variables from the metabolomics data that were useful for the classification of the data set into differential groups of fungal doses. The SDA model made selections based on statistical criteria at the defined significance levels to ‘enter’ and ‘remain’ within the model. In the SDA, the significance level to enter and to stay within the model was set at α ≤ 0.15 for Partial R^2^; this was done in order to accommodate potentially important variables. The SDA was conducted using SAS software (version 9.4, SAS Institute Inc, Cary, NC, USA). The second statistical procedure was a linear discriminant analysis (LDA) used for identification of important variables within each stage class. With the LDA, a set of linear statistical features was applied for the classification of the data, consisting of the continuous independent variables of the metabolomics data and the dose category into which the observations were classified. Percentage error of misclassification of observations in each dose category was also determined to evaluate performance of the discriminant criterion and the covariates used. LDA was conducted using the R software.

For the aflatoxin data, total aflatoxin concentrations (ng/g) and absolute aflatoxin levels (ng/kernel) in the kernels were determined using Equations (1) and (2), respectively. Data normality was improved by logarithm value transformation using Equations (3) and (4) for aflatoxin concentration and absolute levels, respectively. Analysis of variance (ANOVA) between the least square means were determined using the generalized linear model (GLM) in SAS (version 9.4) at α = 0.05.

Total aflatoxin concentrations (AFT) = AFB1 + AFB2 + AFG1 + AFG2(1)

Absolute (Absol) aflatoxin = AFT × weight of kernel(2)

Log AFT = Log10 (AFT + 1)(3)

LogAbsol = Log10 (Absol + 1)(4)

By computing the Pearson correlation in SAS (version 9.4), the correlation between primary and secondary metabolites (i.e., compounds from the metabolite analysis of polar/non-polar fractions and Log AFT) were determined. Significance level was set at α = 0.05.

## Figures and Tables

**Table 1:** Log aflatoxin concentrations in kernels within each of the grain maturity stages.

	Log AFT in Grains ± SD *		
Dose	Milk	Dough	Dent
Control	0.00 ± 0.00 ^a^	0.00 ± 0.00 ^a^	0.00 ± 0.00 ^a^
Low	3.88 ± 0.34 ^b^	3.96 ± 0.44 ^b^	3.67 ± 0.45 ^b^
Medium	3.73 ± 0.59 ^b^	4.31 ± 0.34 ^c,∫^	4.01 ± 0.33 ^c^
High	3.78 ± 0.21 ^b^	4.44 ± 0.38 ^c,∫^	4.12 ± 0.27 ^c^

* Different superscripted letters (a, b, c) in the same column represent aflatoxin concentrations that are statistically different from one another. Log AFT values with ∫ indicate values that are statistical differences (*p* < 0.05) within the same row (dose). AFT = total aflatoxin concentrations; SD = standard deviation.

**Table 2:** Log absolute aflatoxin levels in kernels within each of the grain maturity stages.

	Log Absol in Grains ± SD *		
Dose	Milk	Dough	Dent
Control	0.00 ± 0.00 ^a^	0.00 ± 0.00 ^a^	0.00 ± 0.00 ^a^
Low	2.89 ± 0.34 ^b^	3.08 ± 0.41 ^b^	2.99 ± 0.45 ^b^
Medium	2.73 ± 0.59 ^b,∫^	3.33 ± 0.35 ^c^	3.32 ± 0.33 ^c^
High	2.78 ± 0.21 ^b,∫^	3.50 ± 0.40 ^c^	3.42 ± 0.27 ^c^

* Different superscripted letters (a, b, c) in the same column represent aflatoxin concentrations that are statistically different from one another. Log absolute aflatoxin levels (Log Absol) values with ∫ indicate values that are statistical differences (*p* < 0.05) within the same row (dose). SD = standard deviation.

**Table 3:** Dose stage classification accuracy and error from stepwise discriminant analysis.

	Percentage Classified into Dose Group				
From Dose	Control	Low	Medium	High	Error Counts for Dose
Control	73%	16%	2%	9%	0.27
Low	7%	89%	4%	0%	0.11
Medium	9%	2%	89%	0%	0.11
High	2%	0%	2%	96%	0.04

**Table 4:** Pearson’s correlations of log aflatoxins and identified compounds. GABA = gamma-aminobutyric acid.

Compound	R	*p* Value
1,2-Propanediol	−0.46	<0.0001
Alanine	−0.41	<0.0001
Aminomalonic acid	−0.28	0.0010
Arabitol or Xylitol	0.48	<0.0001
Citric acid	0.36	<0.0001
d-erythro-pentitol	0.12	0.1617
Erythronoic acid	−0.41	<0.0001
Fructose	−0.25	<0.0030
GABA	−0.39	<0.0001
Glyceric acid	−0.40	<0.0001
Glycerol	0.41	<0.0001
Glycine	−0.31	0.0002
Inositol	0.45	<0.0001
Iso-leucine	−0.46	<0.0001
l-threitol	0.35	<0.0001
Malate	0.18	0.0310
Malonic acid	0.17	0.0450
Mannitol	−0.16	0.0520
Myo-inositol	−0.47	<0.0001
Phosphoric acid	0.21	0.0140
Proline	−0.48	<0.0001
Serine	−0.42	<0.0001
Sucrose	−0.30	0.0003
Succinate	0.04	0.6800
Threonine	−0.44	<0.0001
Trehalose	−0.49	<0.0001
Turanose	−0.53	<0.0001
Uracil	−0.39	<0.0001
Valine	−0.45	<0.0001
3,4-dimethylbenzoic acid (xylylic acid)	−0.08	0.3702
Pentadecanoic acid (*n*-pentadecanoic acid)	0.15	0.0800
Hexadecanoic acid (palmitic acid)	−0.08	0.3491
Octadecanoic acid (stearic acid)	0.02	0.8282
11-icosenoic acid (gondoic acid)	−0.15	0.0705
Icosanoic acid (arachidic acid).	−0.03	0.7195

## Supplementary Materials

The following are available online at http://www.mdpi.com/2072-6651/10/5/187/s1, Figure S1: Acid metabolites identified at the milk stage (error bars indicate standard error), Figure S2: Acid metabolites identified at the milk stage (error bars indicate standard error), Figure S3: Acid metabolites identified at the dent stage (error bars indicate standard error), Figure S4: Sugar metabolites identified at the milk stage (error bars indicate standard error), Figure S5: Sugar metabolites identified at the dough stage (error bars indicate standard error), Figure S6: Sugar metabolites identified at the dent stage (error bars indicate standard error), Figure S7: Sugar alcohol metabolites identified at the milk stage (error bars indicate standard error), Figure S8: Sugar alcohol metabolites identified at the dough stage (error bars indicate standard error), Figure S9: Sugar alcohol metabolites identified at the dent stage (error bars indicate standard error), Figure S10: Non-polar metabolites identified at the milk stage (error bars indicate standard error), Figure S11: Non-polar metabolites identified at the dough stage (error bars indicate standard error), Figure S12: Non-polar metabolites identified at the dent stage (error bars indicate standard error), Table S1: Polar analyte statistics for analyte selection from stepwise discriminant analysis.
