# Supplementary material for: Metabolites Identified during Varied Doses of Aspergillus Species in Zea mays Grains, and Their Correlation with Aflatoxin Levels
Source: Toxins (Basel). 2018 May 7;10(5):187. doi: 10.3390/toxins10050187 (PMC5983243; doi:10.3390/toxins10050187)
Supplement: Supplementary file 1 [file toxins-10-00187-s001.pdf]

Supplementary Materials: Metabolites Identified during Varied Doses of Aspergillus Species in Zea mays Grains, and Their Correlation with Aflatoxin Levels

Titilayo D. O. Falade, Panagiotis K. Chrysanthopoulos, Mark P. Hodson, Yasmina Sultanbawa, Mary Fletcher, Ross Darnell, Sam Korie and Glen Fox

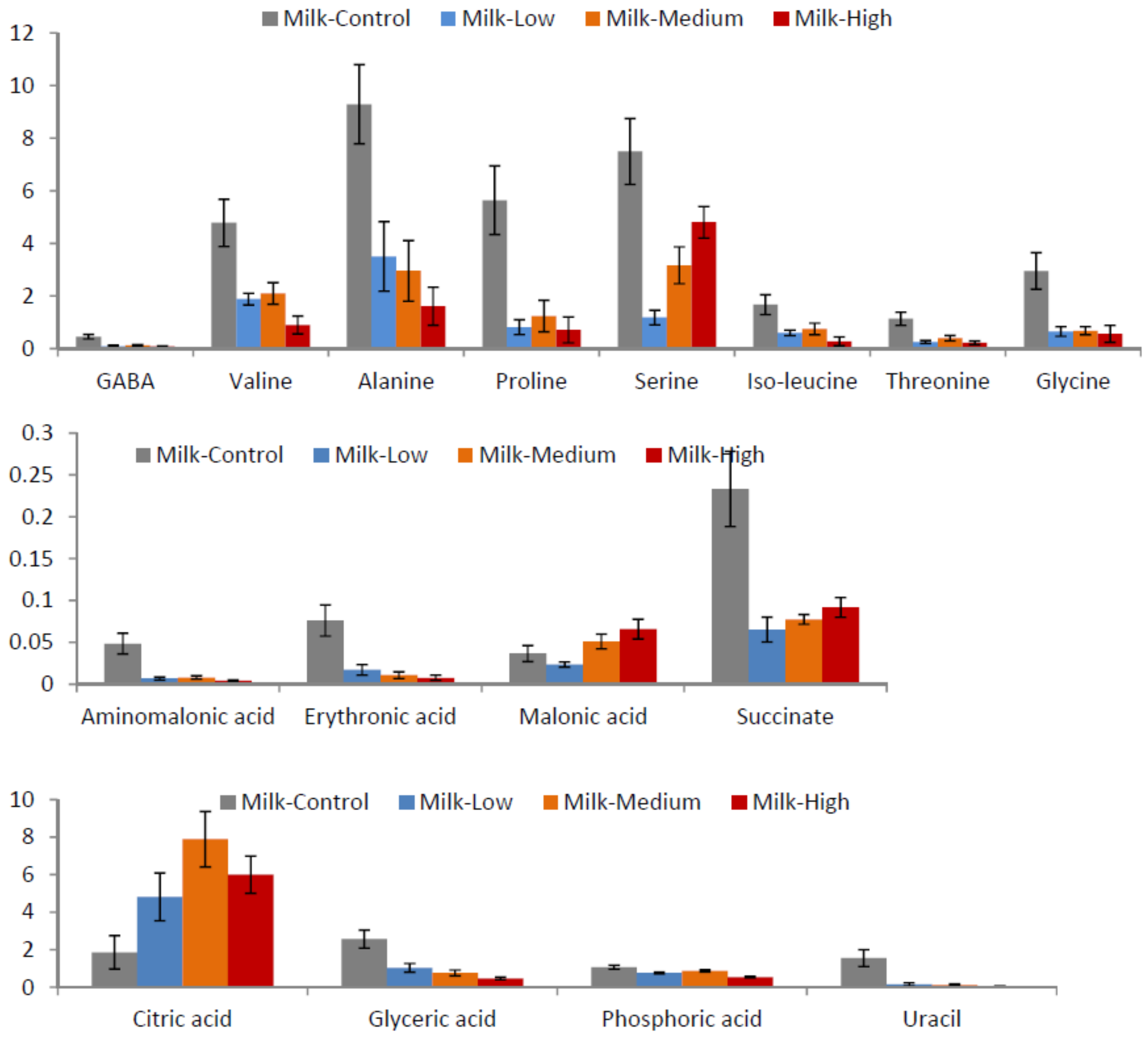

Figure S1. Acid metabolites identified at the milk stage (error bars indicate standard error).

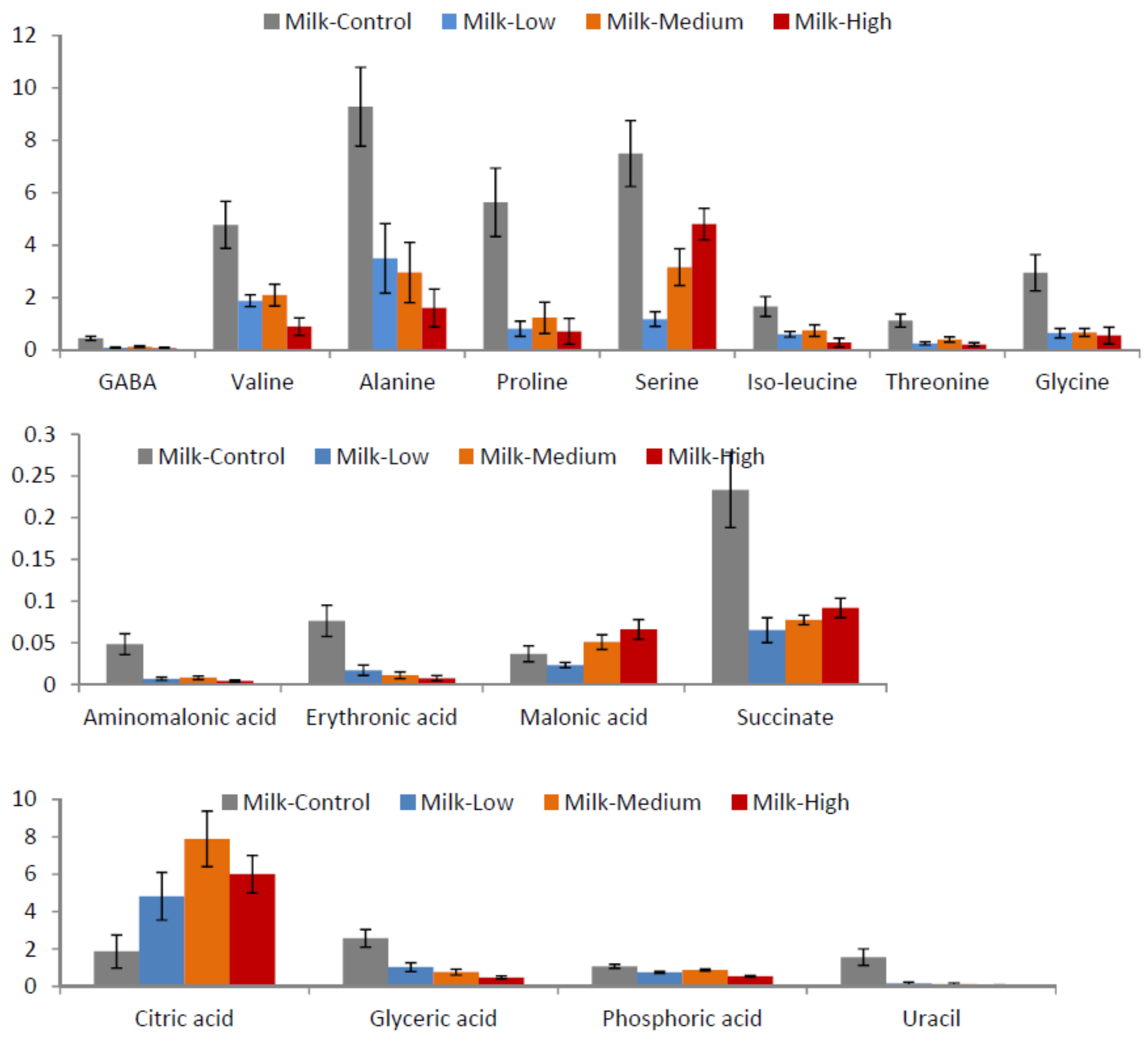

Figure S2. Acid metabolites identified at the milk stage (error bars indicate standard error).

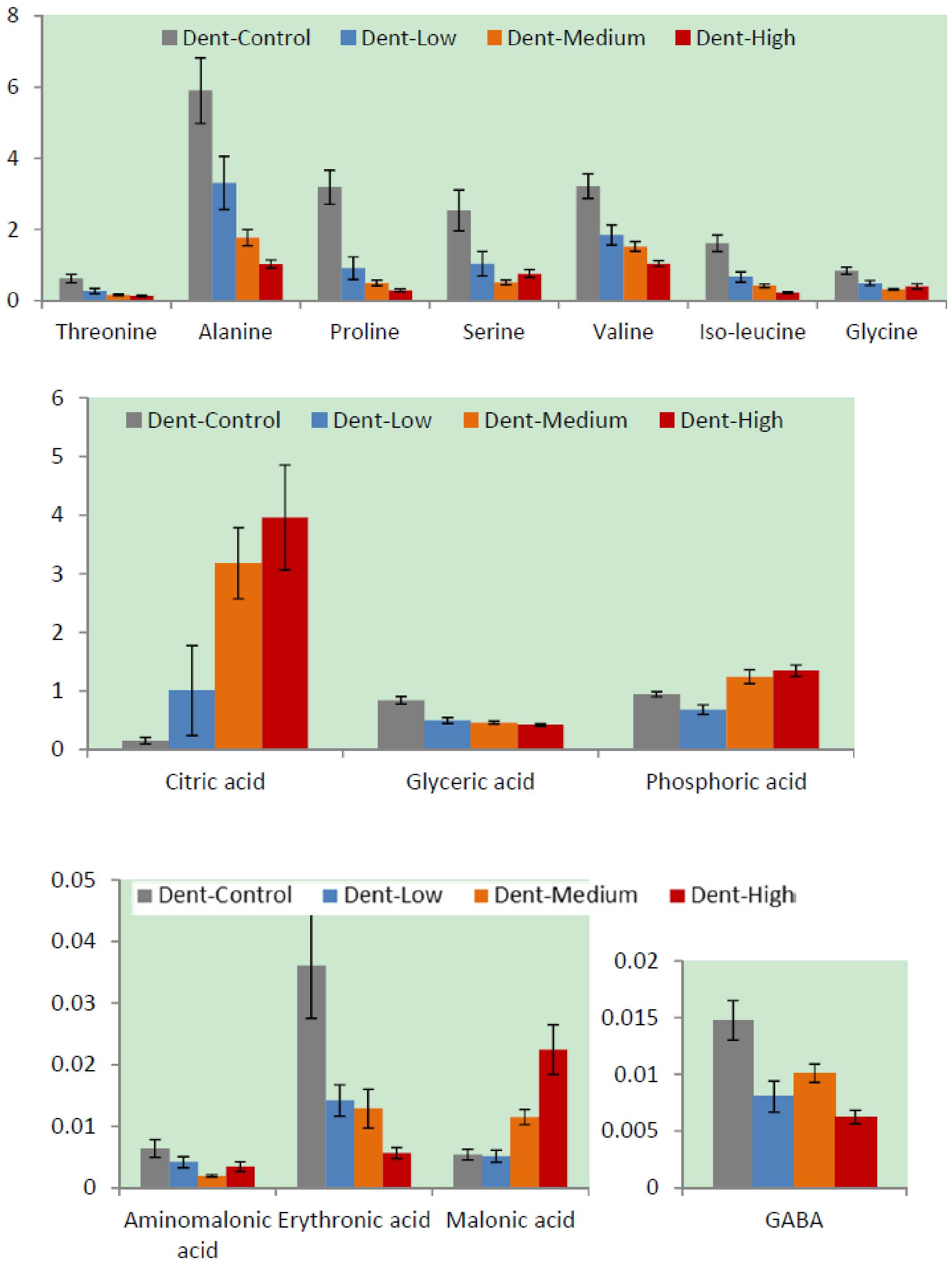

Figure S3. Acid metabolites identified at the dent stage (error bars indicate standard error).

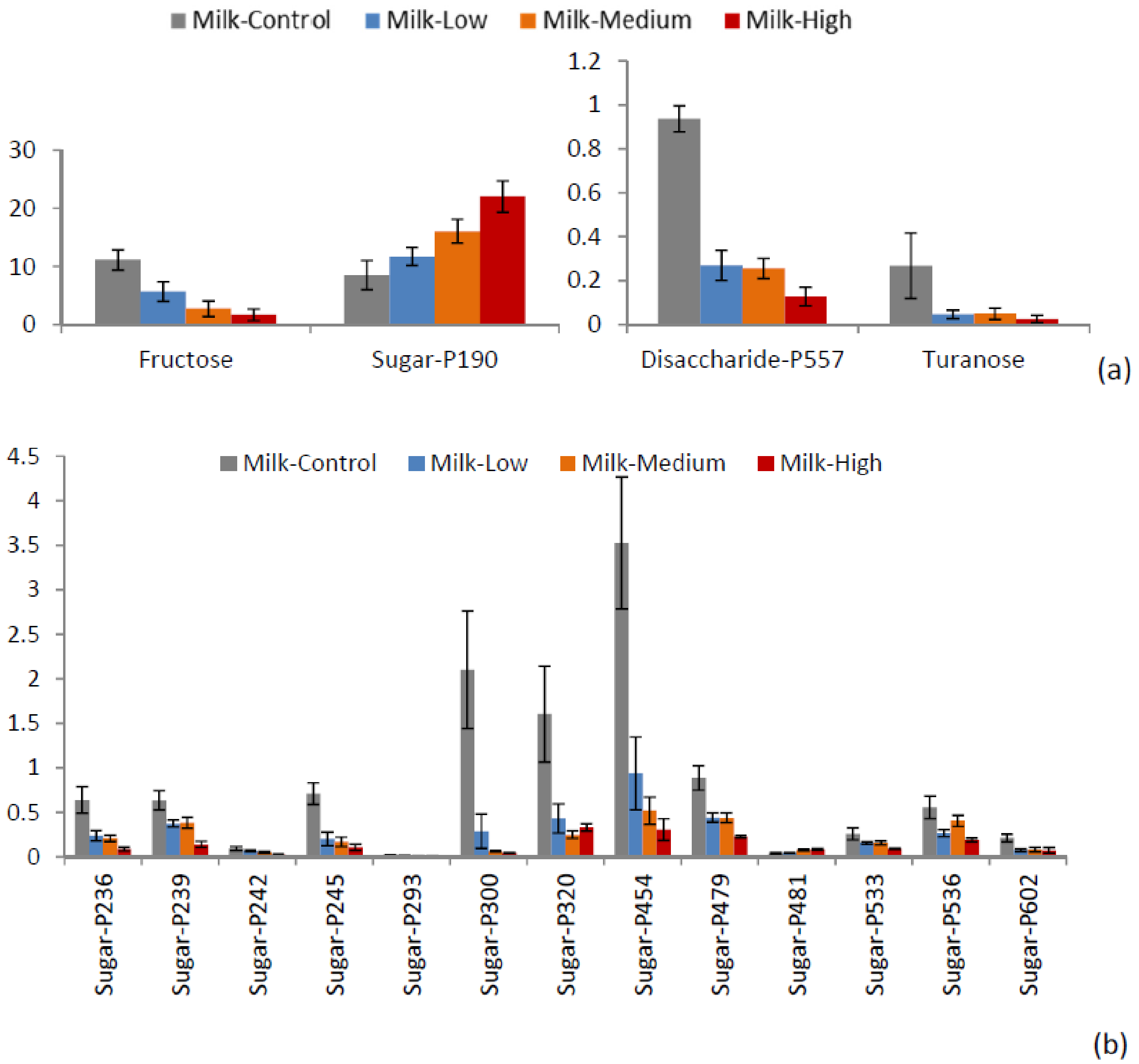

Figure S4. Sugar metabolites identified at the milk stage (error bars indicate standard error).

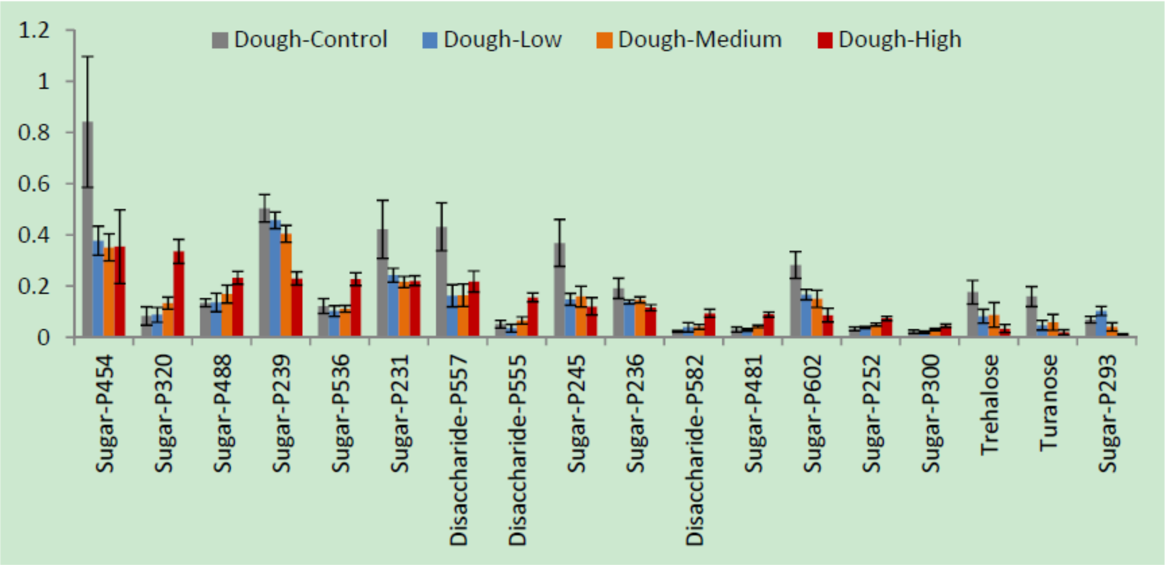

(a)

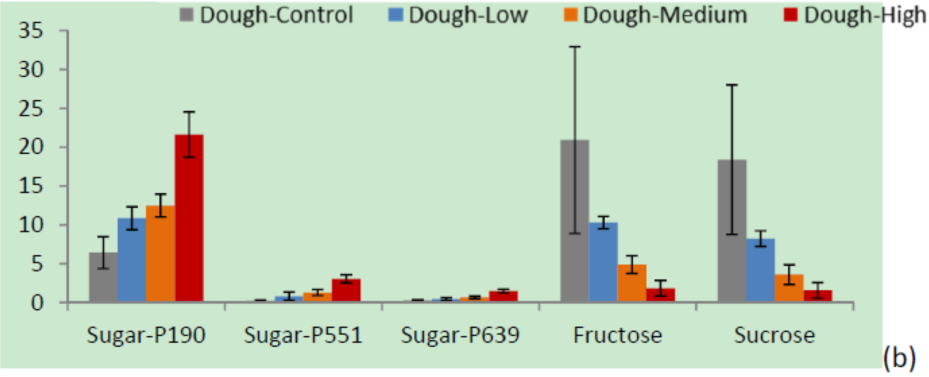

(b)

Figure S5. Sugar metabolites identified at the dough stage (error bars indicate standard error).

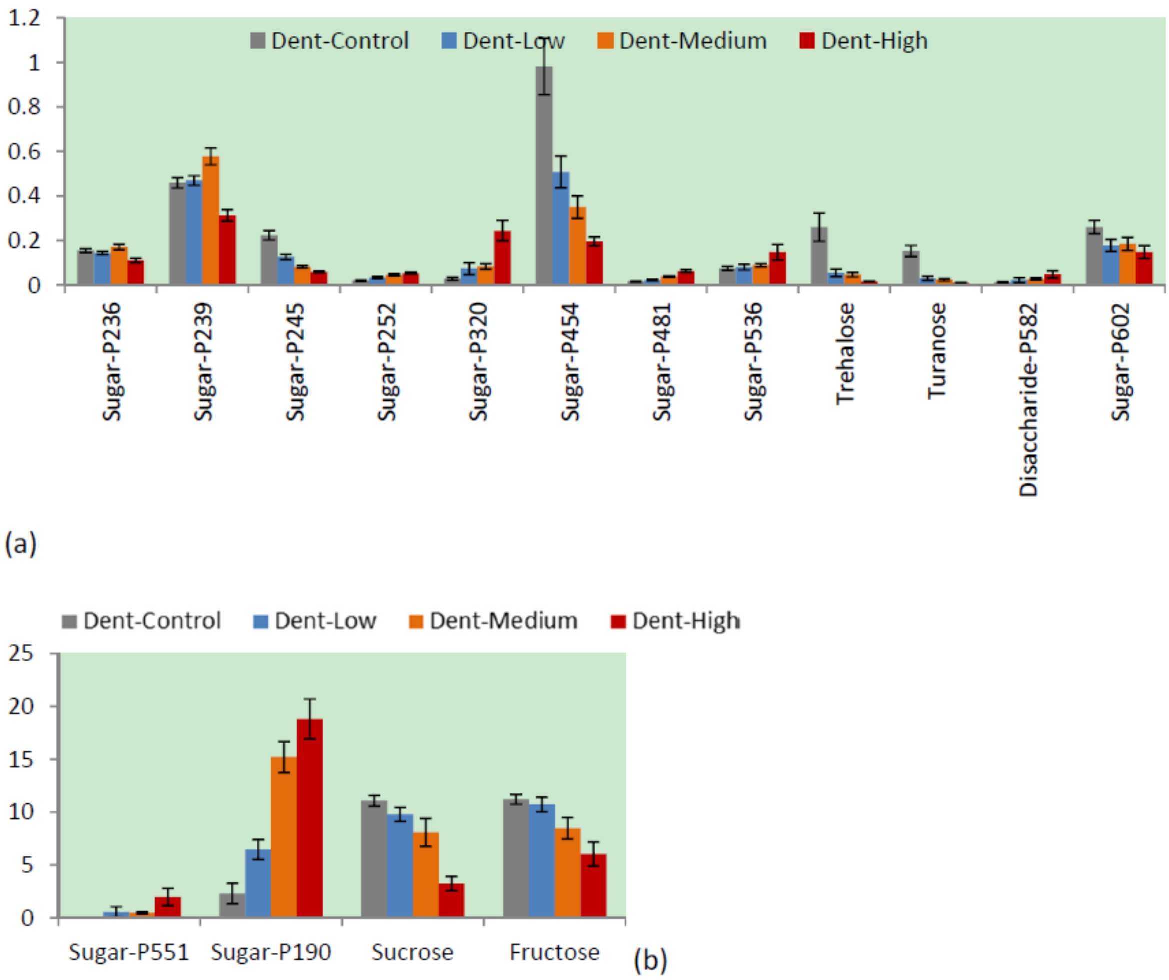

Figure S6. Sugar metabolites identified at the dent stage (error bars indicate standard error).

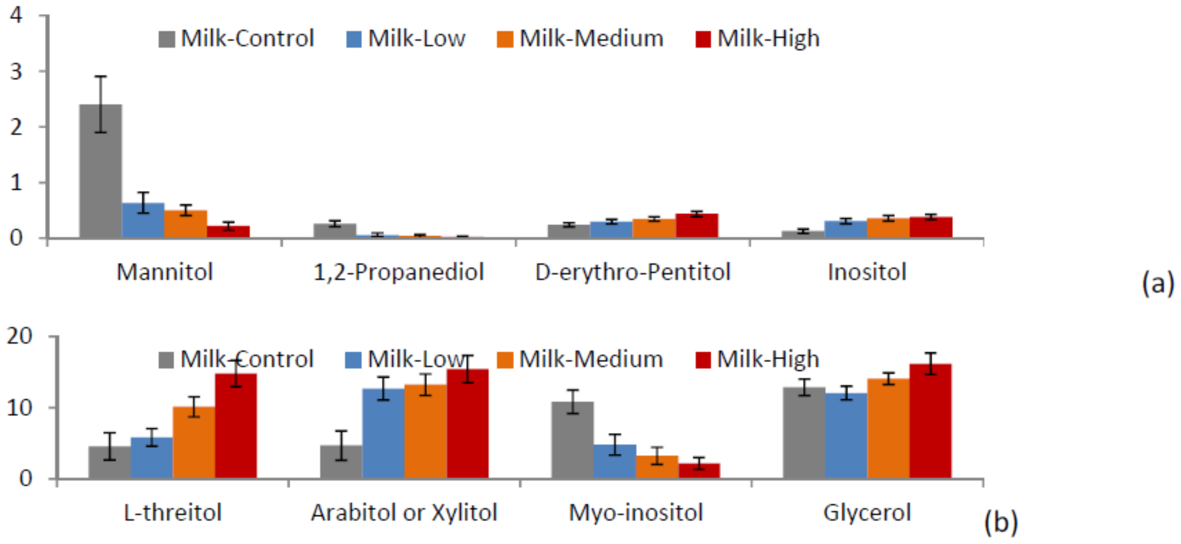

Figure S7. Sugar alcohol metabolites identified at the milk stage (error bars indicate standard error).

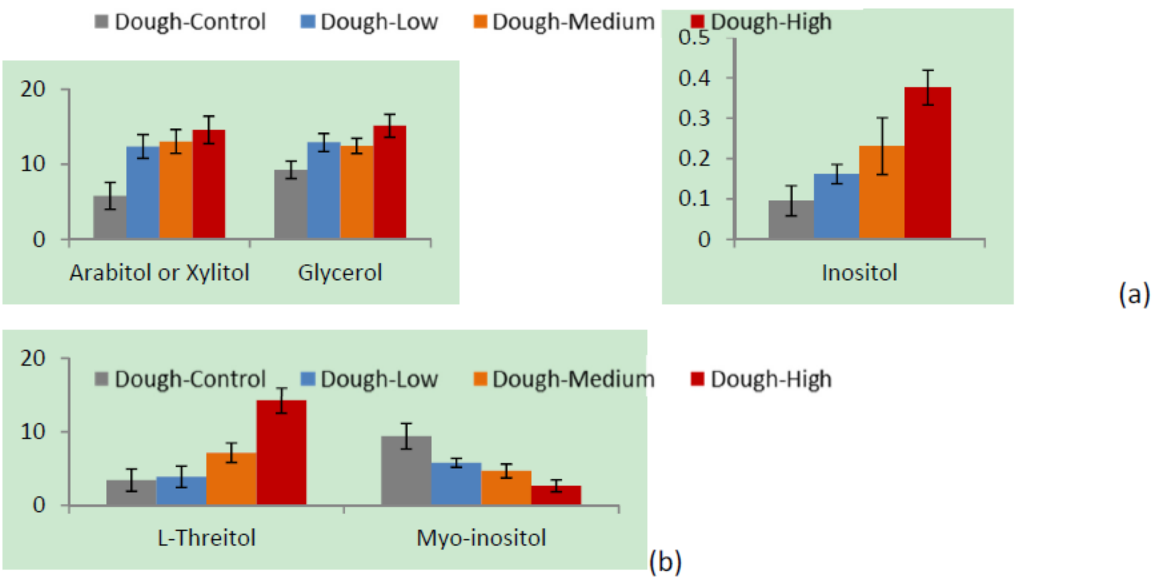

Figure S8. Sugar alcohol metabolites identified at the dough stage (error bars indicate standard error).

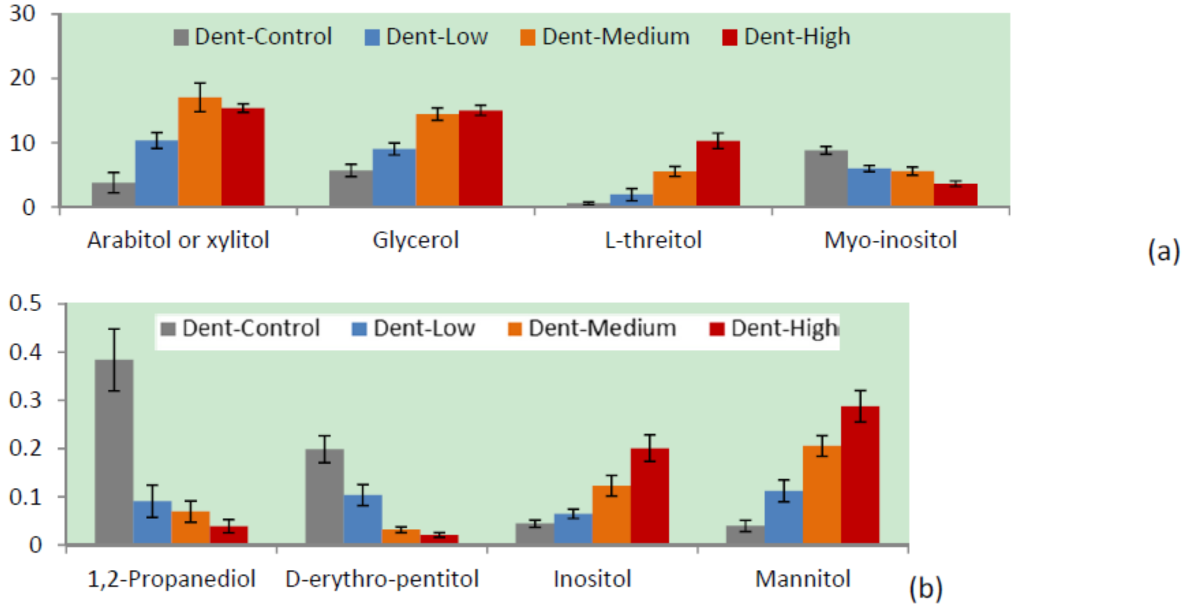

Figure S9. Sugar alcohol metabolites identified at the dent stage (error bars indicate standard error).

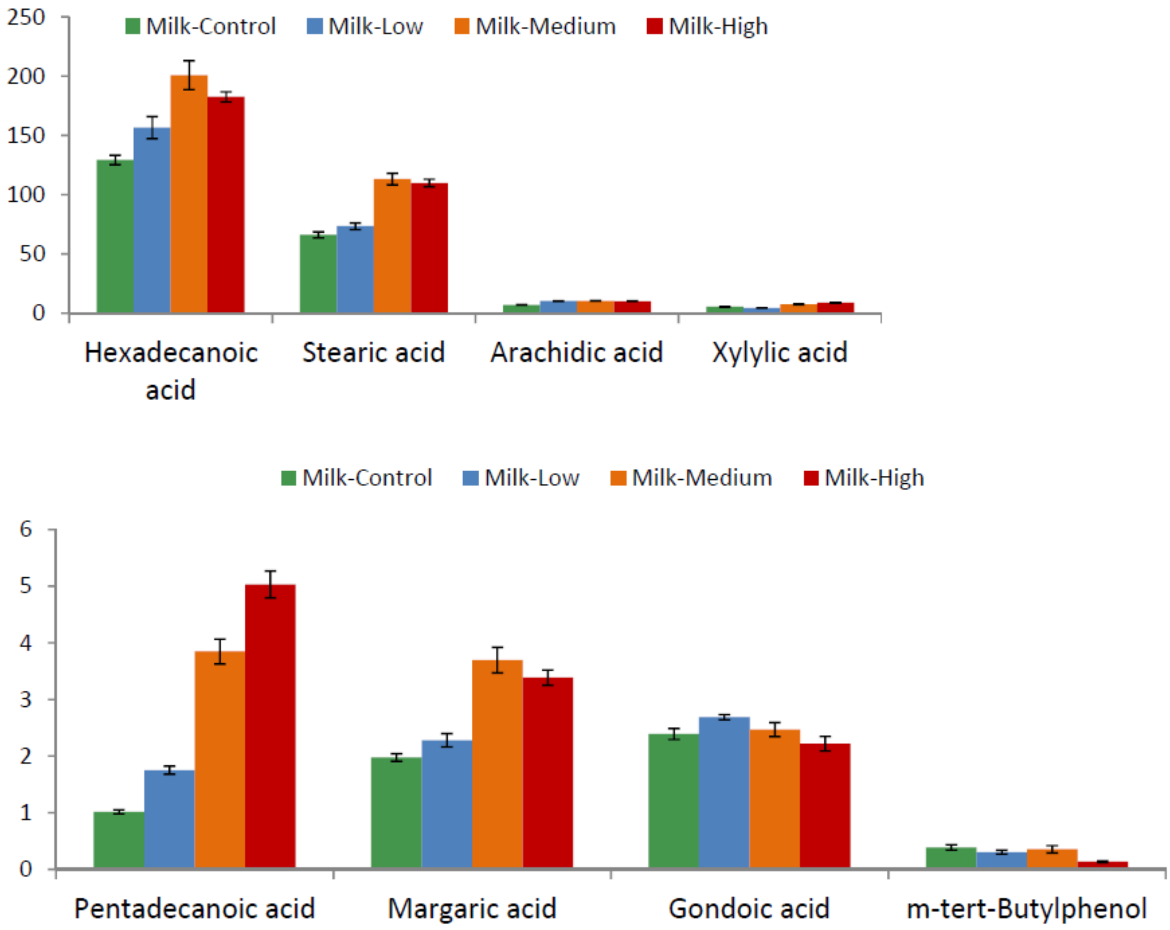

Figure S10. Non-polar metabolites identified at the milk stage (error bars indicate standard error).

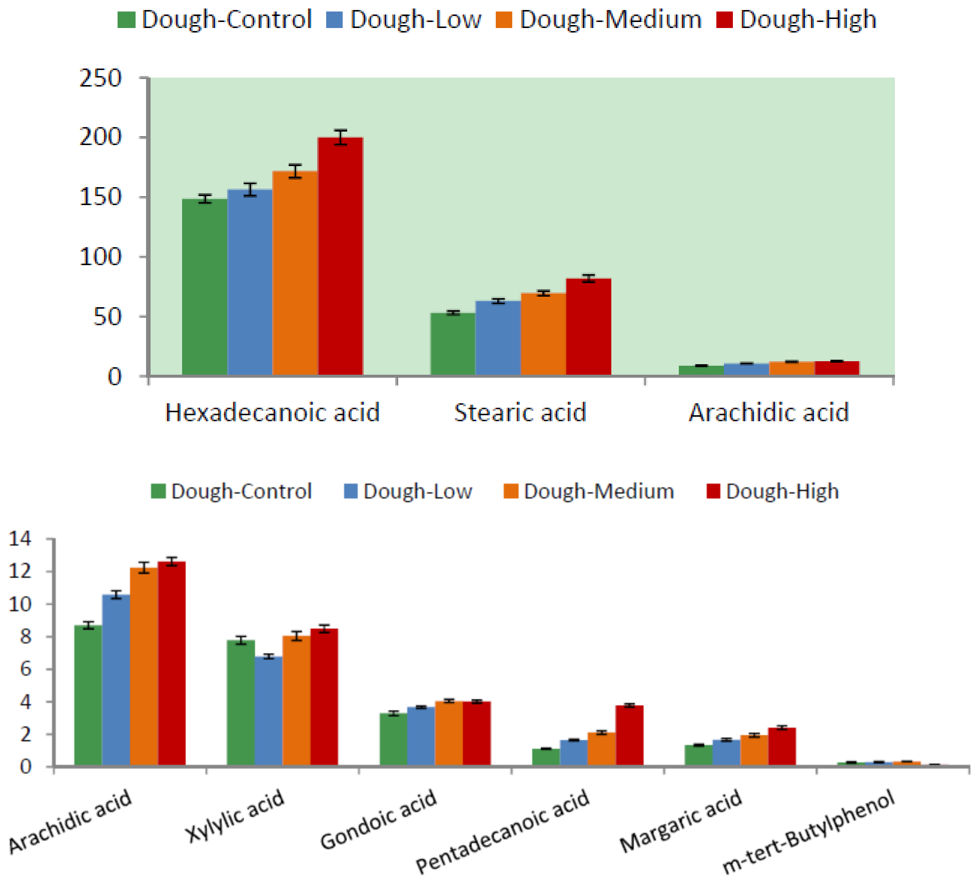

Figure S11. Non-polar metabolites identified at the dough stage (error bars indicate standard error).

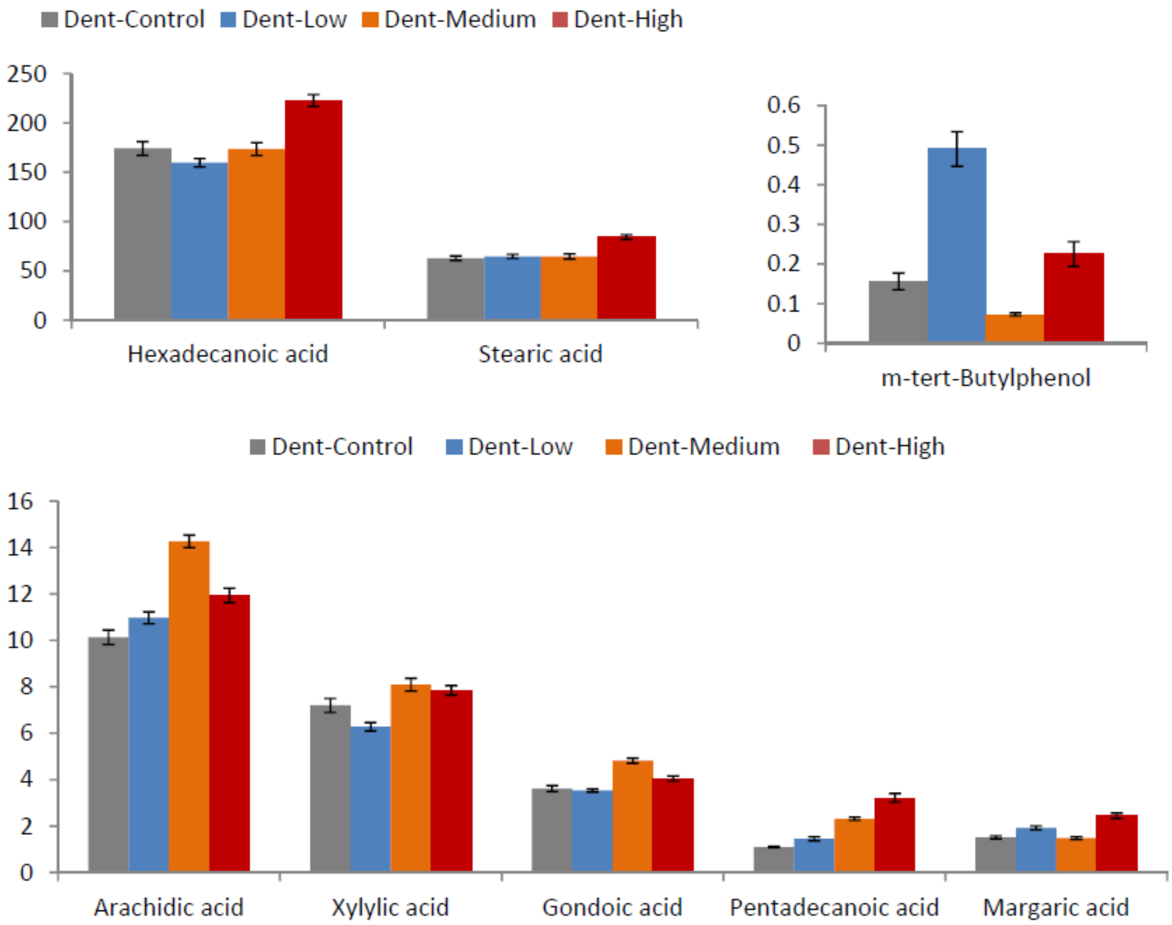

Figure S12. Non-polar metabolites identified at the dent stage (error bars indicate standard error).

Table S1. Polar analyte statistics for analyte selection from stepwise discriminant analysis.

Analyte	Partial R2	F value	P value
Alanine	0.0812	4.59	0.0041
Proline	0.0465	2.42	0.0681
Serine	0.077	4.36	0.0056
GABA	0.0715	4.26	0.0063
Arabitol or xylitol	0.2012	14.61	<.0001
Inositol	0.0964	5.94	0.0007
Malate	0.0611	3.36	0.0203
Glyceryl-glycoside	0.0549	3.16	0.0263
Phosphoric acid	0.0639	3.64	0.0141
Sugar-P231	0.0492	2.78	0.0431
Sugar-P252	0.0779	4.68	0.0037
Sugar-P293	0.0517	2.82	0.0409
Sugar-P481	0.0706	4.25	0.0063
Sugar-P488	0.0481	2.55	0.0582
Sugar-P602	0.0408	2.08	0.1047
Disaccharide-P555	0.0493	2.73	0.0456
Disaccharide-P605	0.0444	2.37	0.073
UK-P73	0.0713	4.28	0.0061
UK-P74	0.0549	2.94	0.0349
UK-P84	0.0692	3.84	0.0109
UK-P179	0.0448	2.35	0.075
UK-P188	0.0656	3.86	0.0105
UK-P209	0.0439	2.27	0.0831
UK-P291	0.0494	2.8	0.0415
UK-P558	0.1722	12	<.0001
UK-P715	0.0567	3.31	0.0216
UK-P725	0.0475	2.56	0.0572
